# Rare retroperitoneal hematoma after percutaneous endoscopic lumbar discectomy: a case report and literature review

**DOI:** 10.3389/fsurg.2025.1503225

**Published:** 2025-04-03

**Authors:** Haiyan Shao, Wenhao Zhu, Xiaochun Xiong, Jie Yu, Zhaoxiang Fan, Chenghong Zhou

**Affiliations:** ^1^ Department of Orthopedics, Zhoushan Hospital of Traditional Chinese Medicine Affiliated to Zhejiang Chinese Medical University, Zhoushan, China; ^2^ Department of Orthopedics, Longhua Hospital, Shanghai University of Traditional Chinese Medicine, Shanghai, China

**Keywords:** percutaneous endoscopic lumbar discectomy, retroperitoneal hematoma, digital subtraction angiography, arterial embolization, case report

## Abstract

**Background:**

Percutaneous endoscopic lumbar discectomy (PELD) has emerged as a first-line surgical option for the management of lumbar disc herniation (LDH). However, postoperative complications remain a concern. We present a rare case of retroperitoneal hematoma (RPH) following PELD.

**Case description:**

A 79-year-old woman who underwent PELD presented with pain in the left inguinal region, lower back, and abdomen, accompanied by hypotension and tachycardia. Abdominal computed tomography (CT) revealed a left-sided RPH. Digital subtraction angiography (DSA) identified a rupture of a left fourth lumbar segmental artery branch. Emergency coil embolization was performed to control the bleeding. Four weeks later, due to the persistence of the hematoma, hematoma evacuation was carried out. Following the procedure, the patient's symptoms resolved, and she experienced relief from discomfort in the left inguinal, lower back, and abdominal regions.

**Conclusion:**

DSA is critical for diagnosing lumbar arterial bleeding, and arterial embolization is an effective approach to hemostasis. Moreover, a comprehensive understanding of the lumbar intervertebral foraminal space anatomy and enhanced surgical techniques are essential to reduce the risk of retroperitoneal hematoma after PELD. Future studies should focus on optimizing the perioperative management process of PELD to enhance the safety of the procedure.

## Introduction

In recent years, with advancements in minimally invasive techniques, percutaneous endoscopic lumbar discectomy (PELD) has become the preferred surgical option for spine surgeons treating LDH. Compared to traditional open spine surgery, PELD offers the advantages of reduced trauma, shorter hospital stays, faster recovery, and the ability to be performed under local anesthesia, making it increasingly and widely accepted by surgeons (1, 2).

PELD is typically categorized based on the approach, into percutaneous endoscopic transforaminal discectomy (PETD) and percutaneous endoscopic interlaminar discectomy (PEID), each with distinct indications (3, 4). Driven by the principles of minimal invasiveness, along with innovations in endoscopic instrumentation and imaging systems, the indications and clinical applications of PELD have gradually expanded. However, complications following PELD have emerged as an issue that cannot be ignored (5, 6). RPH is a rare but serious complication following PELD, with an incidence ranging from 0.12% to 0.97% (7, 8). Reports of such cases in the literature are limited.

Here, we present a case of RPH following PELD, summarize the management process, analyze the possible causes, and review the relevant literature for further reference.

## Case report summary

A 79-year-old woman presented to the Department of Spine Surgery at Zhoushan Hospital of Traditional Chinese Medicine with a 6-month history of recurrent lower back pain, radiating to the left lower limb, which had worsened over the past month. Computed tomography (CT) and magnetic resonance imaging (MRI) of the lumbar spine revealed left L4–5 disc herniation and lumbar 5 nerve root compression without evidence of ossified spinal stenosis (Supplementary Figure 1).

After excluding preoperative contraindications, the patient underwent left L4–5 PETD under local anesthesia (Supplementary Figure 2). The procedure was uneventful. However, 26 h postoperatively, the patient developed distending pain in the left inguinal region, lower back, and abdomen, accompanied by hypotension and tachycardia. Emergency abdominal CT imaging revealed the formation of a left RPH. After an emergency multidisciplinary treatment (MDT) discussion, a lumbar segmental artery injury during the operative procedure was suspected. DSA was performed, which identified a truncated blood supply to the left fourth lumbar segmental artery. Coil embolization was promptly carried out to manage the bleeding. Four weeks later, a hematoma evacuation was performed due to the persistence of the hematoma. The patient's symptoms in the left inguinal, lower back, and abdominal regions resolved, and she was subsequently discharged from the hospital. The complete timeline for the diagnosis and management of RPH in this case is shown in Supplementary Figure 3.

### Past medical history

The patient had a 10-year history of hypertension, which was well-controlled with medication. She also had type 2 diabetes, which was poorly controlled. The patient denied any other significant medical history. On admission, the patient's fasting blood glucose was 11.3 mmol/L (normal range, <6.1 mmol/L), postprandial blood glucose exceeded 17 mmol/L (normal range, <7.8 mmol/L), and her glycosylated hemoglobin level was >8.6% (normal range, 4.0%–6.0%). Due to poor blood glucose control, the patient was transferred to the endocrinology department for optimization of blood glucose levels before surgery. After 10 days of treatment, her blood glucose levels were successfully controlled within the normal range, and she was subsequently transferred back to the Department of Spine Surgery for further preoperative preparation.

### Surgical procedure

The patient's preoperative examination revealed no obvious abnormality. The patient was positioned in the prone position, and a standard PETD was performed. Under C-arm fluoroscopy, a Kirschner wire was used to locate the herniated disc segment, and the puncture trajectory was marked. After routine disinfection and sterile draping, local anesthesia was successfully administered approximately 12 cm laterally to the left of the L4/5 interspinous space. A needle was then inserted through the lateral margin of the L5 superior articular process into the L4/5 intervertebral space. Once the positioning was confirmed, the surgical access was progressively enlarged by sequentially inserting working sheaths. Fluoroscopy was repeated to confirm correct positioning, and a foraminoscope was introduced. Thickening of the ligamentum flavum was noted at the L4/5 level, with compression of the dural sac. Part of the ligamentum flavum was excised using bite forceps to expose the dural sac. The sheath was then advanced to the anterior aspect of the dural sac. After clearing the surrounding annulus fibrosus, the herniated nucleus pulposus was removed. The neural foramen was enlarged, and radiofrequency ablation was performed on the ruptured annulus fibrosus to shrink and seal it, ensuring adequate decompression of the nerve root. The endoscope and working sheath were then removed, and the incision was sutured. The surgical procedure lasted 50 min. During the operation, the patient's obesity and the thick subcutaneous fat layer in the lumbar region increased the difficulty of puncture positioning, leading to a relatively higher number of puncture attempts. The remainder of the procedure proceeded smoothly, with minimal intraoperative bleeding. No active bleeding was observed under endoscopy during the procedure, and no significant bleeding was noted following the removal of the endoscope and working portal. The patient was safely transferred back to the ward postoperatively.

### Postoperative examination

Immediately after surgery, the left lower limb straight-leg raising and strengthening tests were negative, and the symptoms of lower limb pain mostly were almost resolved.

Eight hours postoperatively, the patient experienced a transient drop in blood pressure to 90/60 mm Hg (1 mm Hg = 0.133 kPa), which improved to 116/76 mm Hg after rehydration. Physical examination revealed no significant swelling or tenderness around the surgical incision. The abdomen was soft, without tenderness or rebound tenderness. Both lower limbs exhibited normal movement and strength. Laboratory tests showed hemoglobin of 96 g/L (normal range, 115–150 g/L), hematocrit of 28.2% (normal range, 35.0%–45.0%), erythrocyte count of 3.10 × 10^12^/L (normal range, 3.80–5.10 × 10^12^/L), platelet count (PLT) of 155 × 10⁹/L (normal range, 100–300 × 10⁹/L), and elevated white blood cell count (WBC) of 16.8 × 10⁹/L (normal range, 3.5–9.5 × 10⁹/L).

**Figure 1:**
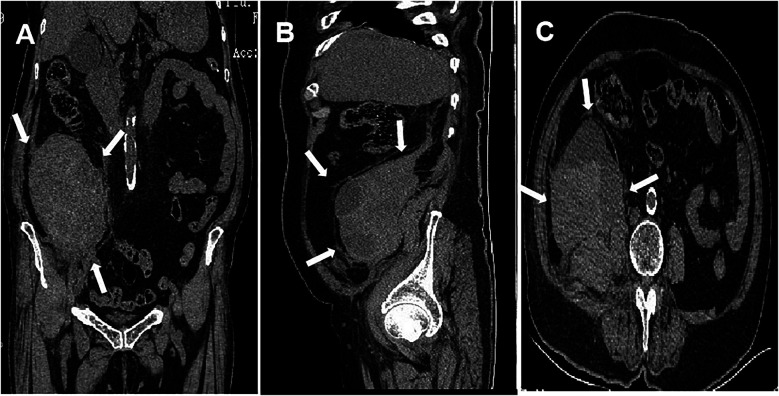
Abdominal CT imaging demonstrating left retroperitoneal hematoma formation (arrows). **(A)** Coronal plane; **(B)** sagittal plane; **(C)** cross-section.

Twenty-six hours postoperatively, the patient's blood pressure again dropped to 90/55 mm Hg, with a heart rate of 112 beats/min. The patient reported distending pain in the left inguinal area, lower back, and abdomen without radiating pain in the lower limb. Physical examination showed mild tenderness in the left inguinal, lower back, and abdominal areas, with no significant swelling or tenderness around the surgical incision. Laboratory findings included hemoglobin of 77 g/L (normal range, 115–150 g/L), hematocrit of 23.7% (normal range, 35.0%–45.0%), erythrocyte count of 2.56 × 10^12^/L (normal range, 3.80–5.10 × 10^12^/L), PLT of 159 × 10⁹/L (normal range, 100–300 × 10⁹/L), and elevated WBC of 27.0 × 10⁹/L (normal range, 3.5–9.5 × 10⁹/L). Coagulation tests revealed a prothrombin time (PT) of 11.6 s (normal range, 9.0–13.1 s), international normalized ratio (INR) of 1.03 (normal range, 0.85–1.15), activated partial thromboplastin time (APTT) of 22.7 s (normal range, 25.4–40.9 s), and thrombin time (TT) of 17.7 s (normal range, 10.3–16.6 s). Abdominal CT revealed the formation of a left RPH (Figure 1).

### Diagnosis and treatment

Based on the symptoms (distending pain in the left inguinal area, lower back, and abdomen), signs (hypotension and tachycardia), and laboratory data (low levels of hemoglobin, erythrocyte pressure, and erythrocyte count), intraperitoneal hemorrhage was considered. The patient received a blood transfusion and vasopressor therapy. Emergency abdominal CT confirmed the presence of a left RPH.

**Figure 2:**
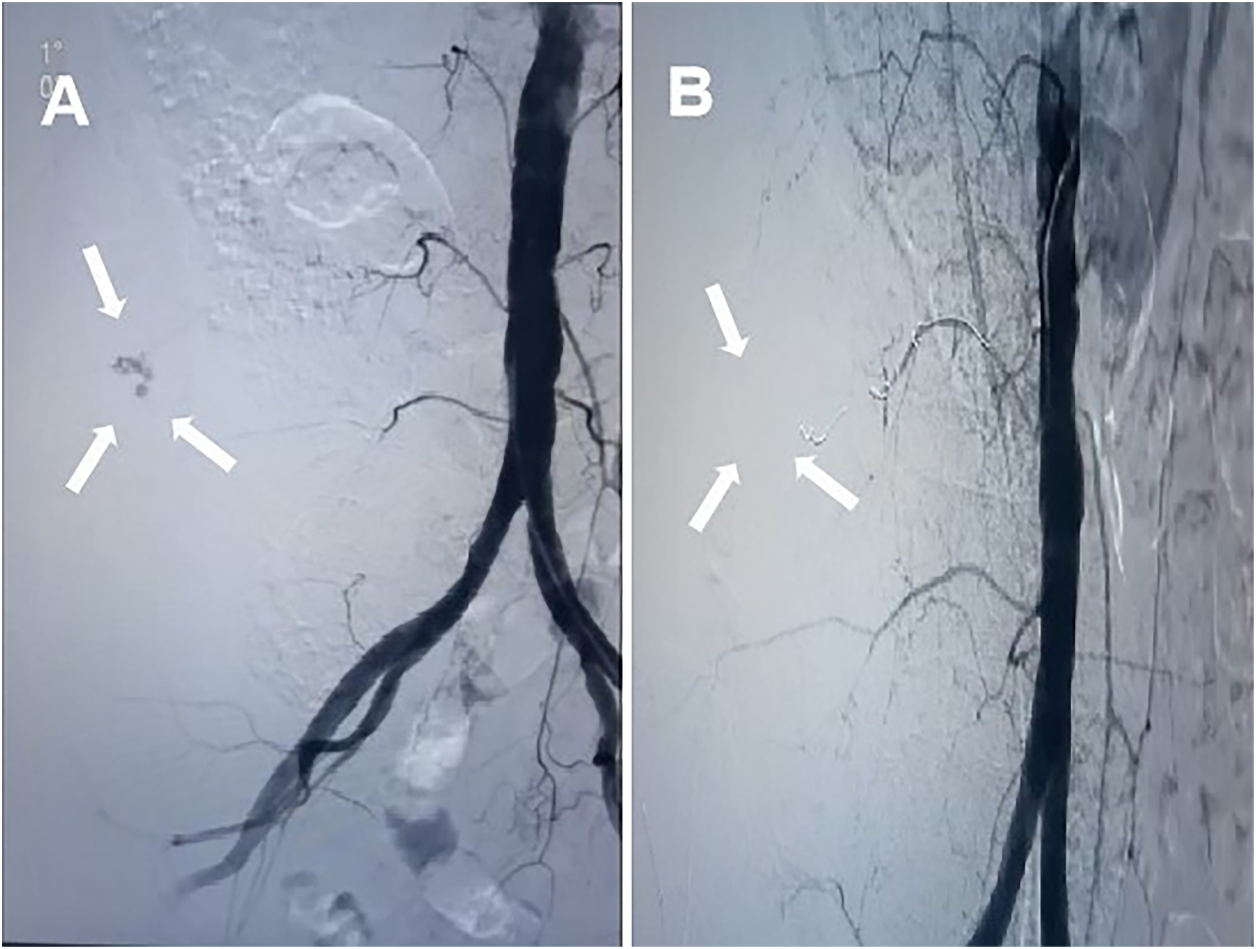
**(A)** DSA showing rupture of a branch of the left fourth lumbar artery (arrows); **(B)** left L4 arterial embolization under DSA with the disappearance of contrast leakage (arrow).

An MDT discussion was held to determine the cause of the hematoma, with injury to the lumbar segmental artery during the surgical procedure considered as a likely etiology. The patient was promptly transferred to the Department of Vascular Surgery for DSA, which revealed truncation of the blood supply to the left fourth lumbar segmental artery (Figure 2A). A contrast leak was identified in the psoas major region at a rate of 0.5 ml/s. Immediate coil embolization was performed, and after 10 min, no contrast leakage was observed at the vascular truncation site (Figure 2B).

### Outcome and follow-up

**Figure 3:**
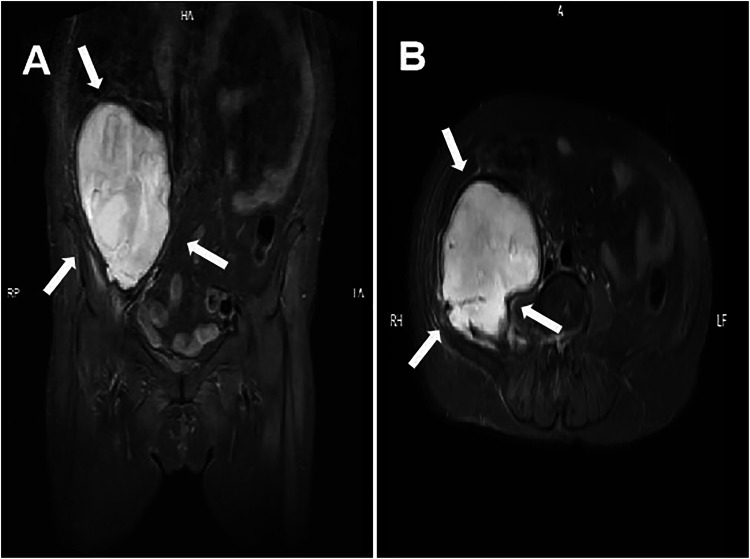
Four weeks after arterial embolization, MRI revealed a retroperitoneal hematoma with minimal resorption (arrows). **(A)** Coronal plane; **(B)** cross-section.

**Figure 4:**
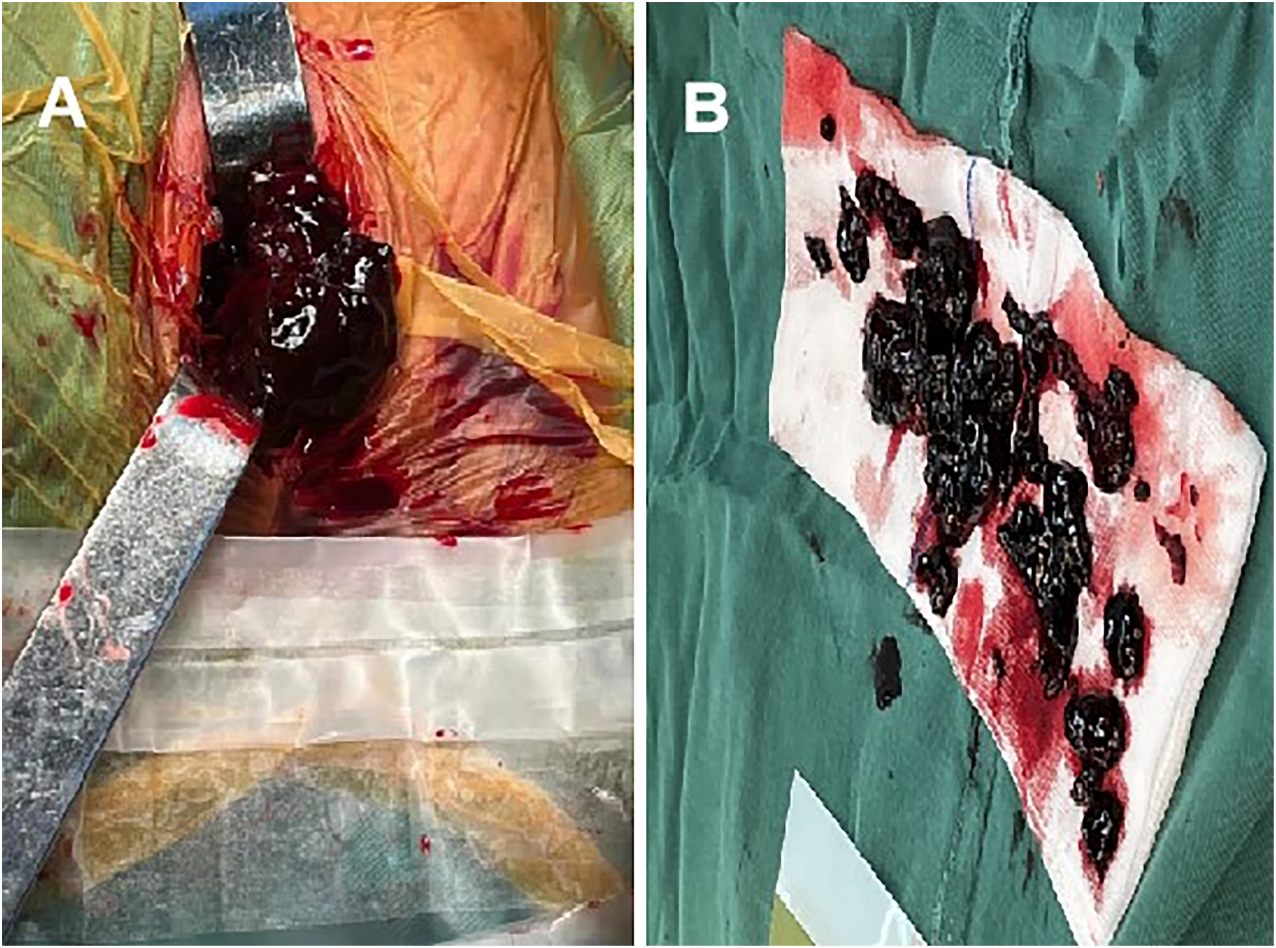
Hematoma removal by open surgery. **(A)** Intraoperative exposure of the hematoma; **(B)** the removed hematoma.

One week after the intervention, the patient's distending pain in the left inguinal area, lower back, and abdomen gradually improved, and there were no neurological symptoms in the left lower limb. Four weeks later, the patient reported mild soreness and discomfort in the left lower back and abdomen. A repeat MRI showed no significant resolution of the hematoma (Figure 3). The hematoma was surgically evacuated through an extraperitoneal incision under anesthesia (Figure 4). Postoperatively, the patient's discomfort in the lower back and abdomen resolved, and she was discharged.

At 6- and 12-month follow-up, the patient had no significant discomfort in the lower back or abdomen.

## Discussion

PETD is a minimally invasive procedure with a low incidence of complications. However, as its application becomes increasingly widespread, reports of complications are gradually increasing. RPH is a rare but serious complication following PETD, with only a few cases reported to date.

Currently, no studies have comprehensively summarized the risk factors for RPH following PETD. It is generally accepted that RPH after PETD primarily results from injury to the lumbar arteries or their branches during the procedure. However, a study by Ahn et al. (7) found that patients with chronic liver cirrhosis or those undergoing repeat PETD procedures may have larger RPH. In terms of diagnosis, RPH typically presents with discomfort in the inguinal region following an asymptomatic period after surgery. Additionally, RPH most commonly occurs after surgery at the L4–L5 segment (Table 1). The anatomical structure of the L4 segmental artery, with its thicker intersegmental artery branches near the intervertebral foramen that travel caudally, makes intraoperative manipulation more challenging (9). Regarding treatment, consensus remains lacking. Ahn et al. (7) suggested that diffuse hematomas (≥500 ml of bleeding) should be managed with open hematoma evacuation, while localized hematomas (bleeding <500 ml) may be treated conservatively. However, Kim et al. (10) reported a case of diffuse hematoma that was treated conservatively. Thus, the decision to operate based on hematoma size is not definitive. Early open surgical interruption of the vessel and hematoma removal is not typically preferred because locating the bleeding point in iatrogenic lumbar artery injuries is often difficult during open surgery. Additionally, retroperitoneal exploration may compromise the encapsulation of the hematoma by peripheral tissues, exacerbating hemorrhage (8). Consequently, early detection and identification of the bleeding point through DSA with subsequent arterial embolization may be the preferred management strategy for RPH (11, 12). While most RPH resolve spontaneously due to the presence of numerous nonstructural cavities in the retroperitoneal region, open surgical evacuation may be necessary for large hematomas (>500 ml) that do not significantly absorb after 3–4 weeks and interfere with the patient's daily life.

**Table 1:** Retroperitoneal hematoma after percutaneous endoscopic lumbar discectomy reported in the literature.

Ref. (arrange by year)	Case	Age, sex	Levels	Time to RPH onset	Clinical features	Treatment	Hematoma volume (ml)	Prognosis
Ahn et al. (7) 2009	4	64, M	L4/5	3 h	Inguinal and low back pain	Open hematoma evacuation	1,274.1	No residual symptoms
		31, F	L4/5	4 h	Inguinal and low back pain	Open hematoma evacuation	704.0	No residual symptoms
		34, M	L4/5	4 h	Inguinal and thigh pain	Conservative	80.2	No residual symptoms
		41, F	L3/4、L4/5	0.5 h	Inguinal pain and hip flexion weakness	Conservative	53.3	Slightly weak muscle power in hip flexion
Kim et al. (10) 2009	1	60, F	L4/5	24 h	Low back pain and hip flexion pain, hypotension	Conservative	Unknown	Mild residual pain in lower extremities
Yörükoglu et al. (8) 2017	1	Unknown	L3/4	Unknown	Unknown	Conservative	Unknown	Unknown
Gioffrè G et al. (11) 2019	1	60, M	L2/3	3 h	Inguinal and low back pain	Arterial embolization	About 600	
Cho et al. (12) 2022	2	31, M	L4/5	17 h	Lower abdominal pain	Arterial embolization	Unknown	
		75, F	L4/5	8 h	Low back pain and poor mental state	Arterial embolization	Unknown	

In this case, the primary cause of RPH following PETD may be related to repeated punctures resulting in injury to the lumbar artery. Specifically, the patient's obesity and thick subcutaneous fat layer in the lumbar region increased the length of the puncture needle's path to the target site, making it more prone to deviation from the intended trajectory and causing positional errors. To ensure accurate placement of the puncture needle into the L4/5 intervertebral space, we made multiple attempts and adjustments, which may have caused injury to the lumbar artery. Additionally, the patient's long-standing hypertension and diabetes contributed to increased vascular fragility, heightening the risk of lumbar arterial injury.

Clinically, the patient presented with typical postoperative inguinal discomfort, which is consistent with previously reported cases in the literature. However, the patient experienced a drop in blood pressure 8 h postoperatively. At that time, due to insufficient recognition, the drop in blood pressure was attributed to the effects of anesthetic agents or the postoperative stress response, and the possibility of RPH was not initially considered, which serves as an important clinical warning. In terms of treatment, we identified the bleeding site through DSA and performed emergency arterial embolization to control the hemorrhage. However, as the hematoma did not resolve, we subsequently performed hematoma evacuation through an extraperitoneal incision under anesthesia.

Based on this case, we analyzed the potential causes of RPH following PETD and summarized corresponding preventive measures: (1) Repeated punctures significantly increase the likelihood of accidental injury to the lumbar arteries and their branches near the foramen. Therefore, improving surgical skills is essential. In cases with obese patients, such as the present case, preoperative consideration of the difficulty in puncture positioning is necessary. The use of three-dimensional navigation technology (13) can enhance the accuracy of positioning. (2) For elderly patients with a long history of hypertension and diabetes, whose vascular elasticity is compromised and who are at higher risk for vascular injury, the risk of RPH should be considered during preoperative planning for PETD. During the surgical procedure, gentle manipulation should be emphasized to avoid excessive force. (3) Anatomical variations in the branches of the lumbar arteries may exist in certain patients, significantly increasing the risk of injury to these branches during PETD. Therefore, we recommend performing lumbar CT angiography (CTA) as a routine preoperative examination for PETD to plan the surgical approach in advance and minimize the risk of lumbar artery and branch injury during the procedure.

Furthermore, early diagnosis is crucial for a better prognosis of this condition. If a patient develops typical inguinal symptoms postoperatively, especially with concomitant hypotension, lumbar arterial rupture and hemorrhage should be strongly suspected. DSA can help locate the bleeding site, and emergency arterial embolization is an effective treatment.

## Conclusion

RPH following PELD is often underdiagnosed or diagnosed belatedly. Postoperative hypotension may serve as an early warning sign, particularly when accompanied by groin pain or discomfort. These symptoms should prompt a high index of suspicion for rupture and bleeding of the lumbar segmental artery or its branches. As such, postoperative blood pressure monitoring should be regarded as a crucial component of PELD postoperative management. In elderly patients or those with multiple chronic comorbidities, preoperative lumbar CTA is recommended. Moreover, DSA is indispensable for diagnosing lumbar arterial bleeding, while arterial embolization is considered an effective hemostatic intervention. Above all, a thorough understanding of the anatomical structures of the lumbar intervertebral foramen and advanced surgical proficiency is essential for minimizing the risk of complications associated with PELD.

## Data Availability

The original contributions presented in the study are included in the article/Supplementary Material, further inquiries can be directed to the corresponding author.
