# Supplementary material for: Rare retroperitoneal hematoma after percutaneous endoscopic lumbar discectomy: a case report and literature review
Source: Front Surg. 2025 Apr 3;12:1503225. doi: 10.3389/fsurg.2025.1503225 (PMC12003379; doi:10.3389/fsurg.2025.1503225)
Supplement: Supplementary file 1 [file Table1.docx]

**Supplementary Figure**

**Supplementary Figure 1** Preoperative MRI showing left lumbar disc herniation (arrows). A: Sagittal plane; B: Cross-section.

**Supplementary Figure 2** Intraoperative Endoscopic Access. A: Anteroposterior position; B: Lateral position

**Supplementary Figure 3** Timeline for the diagnosis and management of RPH

**Supplementary Figure 1**


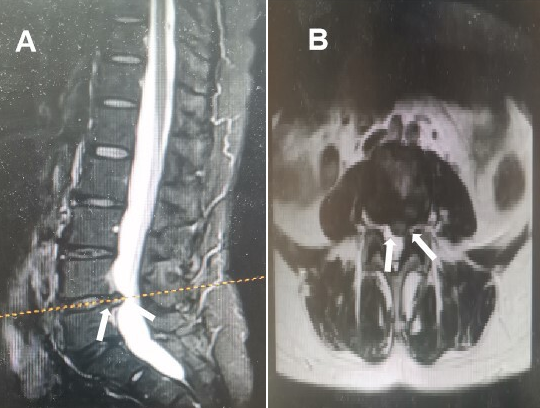


**Figure 1** Preoperative MRI showing left lumbar disc herniation (arrows). A: Sagittal plane; B: Cross-section.

**Supplementary Figure 2**


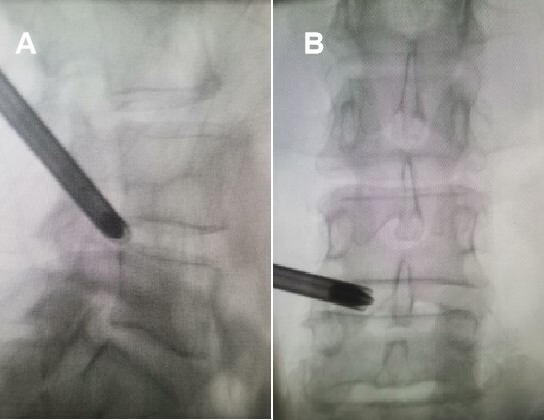


**Figure 2** Intraoperative Endoscopic Access. A: Anteroposterior position; B: Lateral positio

**Supplementary Figure 3**

**Figure 3** Timeline for the diagnosis and management of RPH
